# Use of Oligonucleotides Carrying Photolabile Groups for the Control of the Deposition of Nanoparticles in Surfaces and Nanoparticle Association

**DOI:** 10.3390/ijms12107238

**Published:** 2011-10-24

**Authors:** Brendan Manning, Ramon Eritja

**Affiliations:** 1 Institute for Research in Biomedicine, Baldiri Reixac 10, Barcelona E-08028, Spain; E-Mail: Brendan.manning@gmail.com; 2 Institute for Advanced Chemistry of Catalonia (IQAC), CSIC, CIBER-BBN Networking Centre on Bioengineering, Biomaterials and Nanomedicine, Jordi Girona 18-26, Barcelona E-08034, Spain

**Keywords:** DNA, gold nanoparticles, photolysis

## Abstract

An oligodeoxynucleotide hairpin containing a photolabile 2-nitrobenzyl group in the loop and terminated with a thiol function was prepared. The photocleavage of such a hairpin on gold yields a surface activated with a single stranded oligonucleotide which can be utilised to direct the assembly of nanoparticles conjugated with a complementary strand. Analysis of photocleaved surfaces gives nanoparticle coverage one order of magnitude higher than nonphotocleaved surfaces. This illustrates the ability of photocleavable hairpins to direct the assembly of nanomaterials on conducting materials. The conjugation of the photocleavable hairpin to a gold nanoparticle allows the observation of intermolecular interactions between hairpins linked in different nanoparticles, by comparing the thermal dissociations of a hairpin-nanoparticle conjugates at 260 nm and 520 nm. We have also shown that it is possible to permanently alter the physiochemical properties of DNA-nanoparticles by the introduction of a photocleavable group. Indeed for the first time it has been shown that by exposure to UV light the disassembly of nanoparticle aggregates can be induced.

## 1. Introduction

The use of DNA to form macroscopic aggregates was first demonstrated by Mirkin in 1996 [1]. This method involved conjugating two non-complementary oligonucleotides to gold nanoparticles and mixing them in the presence of a bridging strand partially complementary to both strands; this bridging element causing aggregation of the nanoparticles as visible by a plasmon shift in the visible region. This technique was further advanced to develop electric [2] and scanometric [3] based DNA sensors, such sensors have been shown to be orders of magnitude more sensitive than standard fluorophore based assays.

At the same time Alivisatos developed a method to assemble oligonucleotides into rationally designed dimers and trimers [4]. Since the two seminal works by Alivisatos and Mirkin there has been much work in the study of the properties of gold-nanoparticle conjugates and many methods developed to control their aggregation and designed assembly [5–9].

**Scheme 1:**
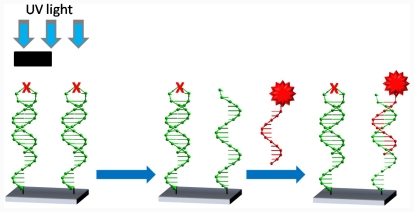
Scheme of a photolithographic process using oligonucleotide hairpins carrying a photolabile group in the loop.

Recently we described the synthesis and properties of oligodeoxynucleotide hairpins carrying a photolabile group in the loop [10]. The modified hairpins have a very stable double-stranded form, as demonstrated by melting experiments. In the presence of the complementary strand, the modified hairpin maintains the entropy-favored intramolecular form. Photolysis of the hairpin generates two single-stranded molecules that have a lower melting temperature than the hairpin [10]. The aim of this work is to link these photolabile molecules onto surfaces to obtain functionalized surfaces. Photolithography of these surfaces results in the cleavage of the oligonucleotides in the illuminated area while in the masked area the oligonucleotides will remain intact (Scheme 1). Utilizing the self-assembling properties of DNA, it is possible to selectively bind nanomaterials to the cleaved biomolecules in the photolyzed areas, as recently demonstrated in silicon oxide surfaces [11].

Here we examine the use of a 3′-dithiol modified photolabile hairpin oligonucleotide to pattern a gold surface. The patterning is achieved by first coupling the oligonucleotide to the surface via a 3′-dithiol functionalisation on the hairpin. Photolysis with UV light of a particular area results in the cleavage of the hairpin and washing leaves only a single stranded oligonucleotide in the irradiated areas. In addition, using the sensitive optical properties of nanoparticle conjugates it is possible to show the existence of aggregates in solution and the photolabile nature of the hairpin is utilized to completely dissociate such aggregates.

## 2. Results and Discussion

### 2.1. Oligonucleotide Synthesis

**Scheme 2:**
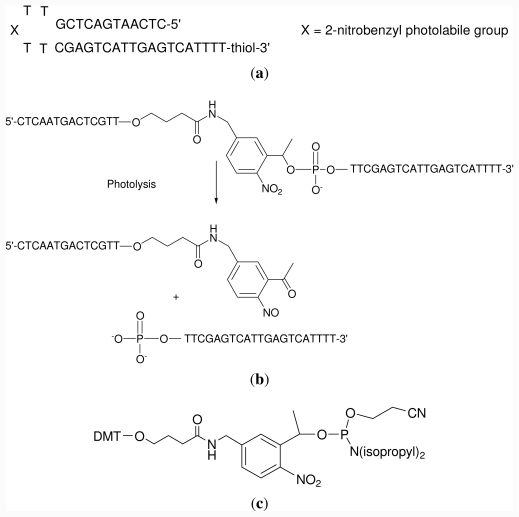
(**a**) Structure of hairpin oligonucleotide A; (**b**) Photolysis of the hairpin oligonucleotide carrying the 2-nitrobenzyl group; (**c**) Phosphoramidite derivative used for the incorporation of the 2-nitrobenzyl group in oligonucleotides.

Hairpin oligonucleotide sequence A was prepared. This oligonucleotide has a photolabile linker in apex of the loop and a dithiol group at the 3′-end. The stable dithiol group at the 3′-end allows direct covalent bonding to a gold surface without the need for cleavage of the protecting group, such as is necessary for an alkyl thiol protecting group. This is advantageous as it is possible to have stable thiol modified hairpin in high concentration for grafting to a surface. The oligonucleotide has a self-complementary sequence linked by a tetrathymidine loop (Scheme 2a). In the middle of the loop there is a photolabile 2-nitrobenzyl group (Scheme 2b). A commercially available phosphoramidite was used for the introduction of the photolabile 2-nitrobenzyl group at the half of the sequence (Scheme 2c).

After ammonia deprotection, the resulting oligonucleotide was purified by reverse-phase HPLC [10]. A thiol terminated complementary oligonucleotide (sequence B, 5′-TGACTCAATGACTCG-propyl-*SS*-propyl-OH-3′) was also synthesized. This was used for the functionalization of gold nanoparticles with the complementary sequence.

### 2.2. Immobilization of Photocleavable Hairpin to Gold Substrates

The grafting of the thiolated photocleavable hairpin A to template stripped gold substrates [12] was monitored by Atomic Force Microscopy (AFM). The extremely flat nature of the substrates makes it ideal to study with AFM. A typical sample of a freshly cleaved substrate has a low Root Mean Square (RMS) roughness of 0.205 nm (Figure S1, supplementary section).

To modify, a template stripped gold surface hairpin A is dissolved in a high ionic strength buffer to minimize electrostatic interactions between DNA strands and allow a high surface density of DNA molecules on the surface. AFM image of surfaces modified with hairpin oligonucleotides show the appearance of small features below 2 nm and a concomitant increase in RMS roughness to 0.390 (Figure S2, supplementary section). These features can be attributed to the DNA or layers of DNA on the surface. Also seen is the disappearance of clear boundary steps between the gold crystal boundaries; these boundaries have been smoothed by the filling-in effect of the DNA covalently bound at the boundary area. The troughs are attributed to defects common in template stripped gold surfaces [13].

**Figure 1:**
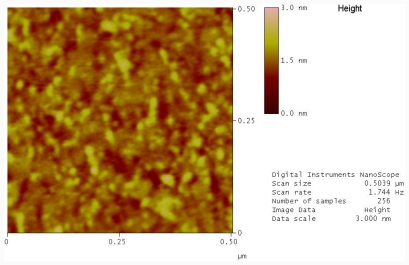
Atomic Force Microscopy (AFM) image of hairpin oligonucleotide on a template stripped gold surface after the addition of 6-mercaptohexanol.

After attachment of oligonucleotide, the surface is exposed to a passivating solution of mercaptohexanol, which has been reported to increase the hybridization efficiency by pushing the oligonucleotide into a more upright position [14,15]. Figure 1 shows an AFM image of a typical mecaptohexanol treated template stripped gold surface. We can see there is a slight increase in the feature sizes, indicating that indeed the DNA is in a more upright position and many of the through like features have disappeared as a result of the mercaptohexanol binding to the surface. This also results in a change of the RMS roughness of the sample to 0.254. From the cross sectional analysis of the image we can see features below 1 nm, typical of a dense DNA layer on gold [16].

### 2.3. Photolysis of Hairpin Oligonucleotide A on a Template Stripped Gold Substrate

**Figure 2:**
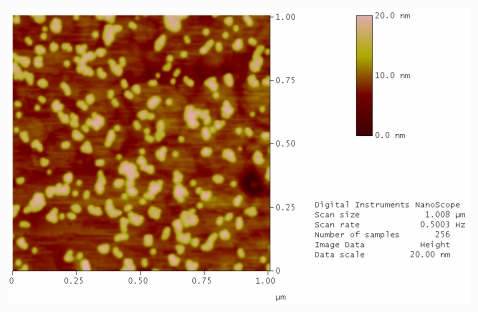
AFM image of template stripped gold surface conjugated with thiolated photocleavable hairpin oligonucleotide, after photolysis with UV source for 30 min and incubation with complementary conjugated gold nanoparticles.

**Figure 3:**
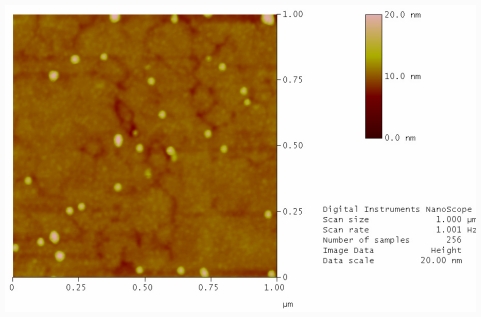
AFM image of template stripped gold surface conjugated with thiolated photocleavable hairpin oligonucleotide and incubation with complementary conjugated gold nanoparticles.

Oligonucleotides were grafted to two freshly cleaved gold surfaces. One substrate was exposed to UV light to cleave the hairpin and one was not. Both samples were then washed with ultra high purity (UHP) water at 90 °C followed by incubating with a solution of complementary oligonucleotide labeled gold nanoparticles. Figure 2 shows a typical AFM image of a gold sample exposed to UV light and complementary labeled gold nanoparticles. As expected, after photocleavage there is an availability of free single stranded oligonucleotides on the surface that can readily hybridize with the nanoparticle conjugates. Particles are lying directing on the DNA layer in a densely packed but statistical distribution. In contrast, the non-photocleaved sample contains the hairpin structure. In the hybridization buffer at room temperature the formation of the hairpin protects the complementary region and the functionalized nanoparticles cannot hybridise to the surface. Therefore, the immobilization of nanoparticles on the surface is scarce (Figure 3).

**Figure 4:**
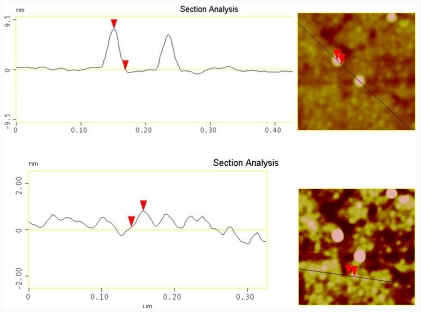
Line analysis of nanoparticles (**above**) and DNA layer (**below**) on a gold substrate with hairpin oligonucleotide, with no photolysis and incubation with complementary conjugated gold nanoparticles.

From a line scan analysis of a sample area from Figure 3 it is possible to see the spherical nanoparticles with a height of 9.5 nm (Figure 4, above) in good agreement with the stated size from the manufacturer (10 nm). It was also possible to see free DNA layer with size of about 1.5 nm below the nanoparticle layer (Figure 4, below).

At this stage it would be desirable to be able to compare the coverage of nanoparticles between photocleaved and non-photocleaved surfaces. The amount of material present was assessed by image analysis. This can be achieved by taking the two images of identical size and converting to 8 bit grayscale images. A thresholding function was used to remove background noise ensuring only material above 8 nm was included in analysis. The resulting images are shown in the supplementary section. The ratio of white pixels to the total number of pixels was evaluated using a histogramming function giving a value for the area covered by nanoparticles. The value was halved to account for AFM tip broadening. It shows that there was a 1.3% surface coverage on the non-photocleaved sample. While on the photocleaved sample the nanoparticle coverage was 17.7%.

### 2.4. Thermal Dissociation Analysis Hairpin Oligonucleotide A Linked to Gold Nanoparticles

**Figure 5:**
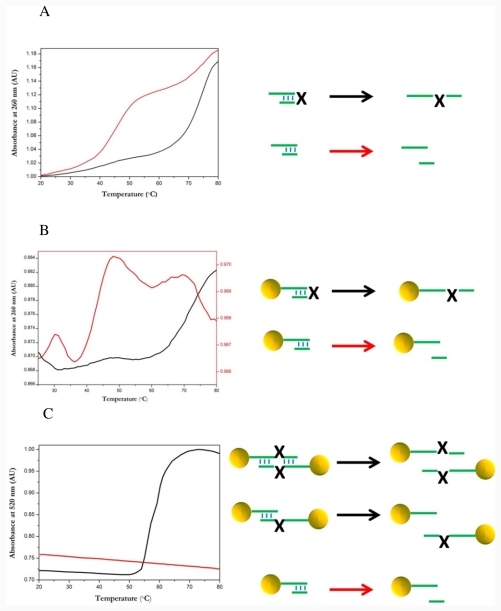
Thermal dissociation curves. (**a**) Photolabile hairpin A measured at 260 nm (Black: before photolysis. Red: after photolysis); (**b**) Gold nanoparticles conjugated with photolabile hairpin A measured at 260 nm. (Black: before photolysis. Red: after photolysis); (**c**) Gold nanoparticles conjugated with photocleavable hairpin A measured at 520 nm. (Black: before photocleavage, Red: after photocleavage). Buffer conditions: 0.3 M NaCl, 0.01% sodium azide, 10 mM sodium phosphate buffer pH 7.1.

In order to further analyze the specific and non-specific binding interactions between photocleavable hairpins and complementary oligonucleotides, gold nanoparticles were functionalized with hairpin oligonucleotide A. Thermal dissociation of hairpin-gold nanoparticle conjugates was investigated at 260 nm and 520 nm. Hairpin-gold nanoparticle conjugates were dissolved in 0.3 M NaCl, 10 mM sodium phosphate buffer, at room temperature thus forming a majority of internal hairpin loops on the nanoparticle. The UV absorbance was recorded at 260 nm while the solution was heated with a temperature gradient of 1 °C/min and a melting curve was plotted, Figure 5B. Two transitions are observed on the melting curve, black line. The first transition is at 75 °C and is primarily due to the denaturation of the hairpin on the nanoparticle. There is also a minor transition at 45 °C due to the presence of some photolysed hairpin. Following this the sample was photolysed by exposure to a strong UV source (black-eye lamp) for 30 min. A second melting curve was plotted as detailed above, Figure 5B. Here there is a major transition at 45 °C corresponding to the photolysed double stranded oligonucleotide and there is no transition observed at 75 °C. The decrease in adsorption after the peaks is caused by loss of solvent from the sample, as the sample is being heated, the evaporated solvent condenses on the lid of the cuvette, since the volume of solvent is quite low this condensation can slightly affect the plots of adsorption *versus* temperature.

Following this the thermal dissociation curves were recorded as described above at 520 nm. At 520 nm we observe absorbance contributions due to a change in the plasmon band of the gold related the average inter-particle distance. In principle we did not expect to see a transition because no intermolecular crosslinking was expected. However a clear transition at about 60 °C was observed in the melting curve, Figure 5C, black curve. This “melting” is caused by the dissociation of hairpin-nanoparticle networks caused by inter-particle hybridization between hairpin strands. The formation of such networks is due to a small amount of open hairpin in solution and a small amount of photolyzed hairpin that allow cross linking between particles. As the temperature is increased individual thermal dissociations can occur between the cross linking strands; however only when all the strands have dissociated are the particles able to move apart. Hence the sharp optical melting, which reflects a change in the average inter particle distance. During the hybridization process there is no notable change in the solution color, indicating that large nanoparticle aggregates are not forming. Following this the hairpin is photocleaved by exposing the sample to a high intensity UV source for 30 min, after which a second thermal dissociation is recorded, Figure 5C, red curve. The derived melting curve shows no transition, hence when photocleaved there is no inter-particle crosslinking due to hairpins because of the lack of complementary sequences between particles.

As described previously DNA-nanoparticle conjugates show significant spectral changes in the UV visible adsorption spectrum at 260 nm, and 520 nm. These include sharper transitions at which larger absorbance changes occur compared to free DNA [1,3].

By comparing the denaturation behavior of hairpin-nanoparticle conjugates at 260 nm and 520 nm it is possible to elucidate some important facts. Firstly the denaturation at 260 nm shows a single broad transition, indeed almost identical to the transition seen for the hairpin without gold nanoparticles, see Figure 5A. Thus indicating the lack of large DNA-nanoparticle aggregates that are commonly seen when complementary DNA-nanoparticles are mixed that is typified by a sharp melting at 260 nm. The lack of such a transition indicates the effectiveness of the self protecting nature of the hairpin. However when the denaturation is observed at 520 nm, a region where transitions are only due to change in the plasmon band of the nanoparticles caused by inter-nanoparticle spacing, there appears a single sharp transition. The only explanation for such a band is that indeed there is some inter-particle crosslinking caused by the hairpins; this crosslinking was not visible in the thermal dissociation recorded at 260 nm due to its low concentration in comparison to the other DNA species. Thus we have shown that it is possible to demonstrate the existence of a minor DNA interstrand interaction by comparison of the thermal dissociation of hairpin-gold nanoparticles at 260 nm and 520 nm. Also demonstrated is the effectiveness of a photolabile modified hairpin to allow control of the physiochemical properties of hairpin-nanoparticles in solution. This is highlighted by the disassembly of the hairpin-nanoparticle aggregates when exposed to UV light. The control of the formation of DNA-mediated nanoparticles aggregates by photocleavage of DNA may be used for the characterization of other DNA-mediated nanocrystal aggregation

## 3. Experimental Section

### 3.1. General

Phosphoramidites and ancillary reagents used during oligodeoxynucleotide synthesis were from Applied Biosystems (PE Biosystems Hispania S.A., Spain). The photolabile phosphoramidite was from Link Technologies (Link Technologies Ltd., Scotland). The rest of the chemicals were purchased from Aldrich, Sigma or Fluka (Sigma-Aldrich Química S.A., Spain). Gold evaporated on mica (template stripped gold) was supplied from Xavier Sisquella from PCB (Parc Científic de Barcelona). Ultra high purity (UHP) H_2_O was purified using a UHQ filtration system and used with a resistivity >18 Ω cm^−1^. 10 nm gold nanoparticles were from *Sigma*.

### 3.2. Oligodeoxynucleotide Synthesis

The hairpin oligonucleotide sequence A, 5′-CTC AAT GAC TCG TT-photolabile-TTC GAG TCA TTG AGT CAT TTT T-propyl-*S*-*S*-propyl-OH-3′ was prepared [10]. This oligonucleotide contains a photolabile linker in the apex of the loop and a thiol group at the 3′-position to attach the oligonucleotide to gold surfaces. The complementary oligonucleotide sequence, B 5′-TGA CTC AAT GAC TCG-propyl-*S*-*S*-propyl-OH-3′ contains a thiol group at the 3′-position to allow coupling to gold nanoparticles. Oligonucleotides were prepared using solid-phase methodology and 2-cyanoethyl phosphoramidites as monomers. The photolabile phosphoramidite (PC spacer-CE phosphoramidite, *Link Technologies*) was used to introduce the photolabile function. For the introduction of the thiol group at the 3′-end we used 3′-thiol-modifier CPG (*Glen Research*) that is a CPG support functionalized with hydroxypropyldisulfide protected with the dimethoxytrityl (DMT) group was used. The syntheses were performed on an *Applied Biosystems* Model 3400 DNA synthesizer using 0.2 and 1μmol scales. After the assembly of sequences, ammonia deprotection was performed overnight at 55 °C (sequence B). Oligonucleotides were purified by reverse-phase HPLC. HPLC solutions were as follows. Solvent A: 5% acetonitrile in 100 mM triethylammonium acetate (pH 6.5) and solvent B: 70% acetonitrile in 100 mM triethylammonium acetate pH 6.5. Columns: Nucleosil 120C18 (10 μm), 200 × 10 mm. Flow rate: 3 mL/min. Conditions A (DMT on): 20 min linear gradient from 15–80% B. Conditions B (DMT off): 20 min linear gradient from 0–50% B. Mass spectrometry: Sequence A (MALDI): Found 11563; expected 11566. In addition we observed a peak corresponding to the fragmentation of the oligodeoxynucleotide during the acquisition of the spectra (3′-fragment, found 7033 expected 7044). Sequence B (MALDI): Found 4797; expected 4796.

### 3.3. Conjugation of Thiolated Oligonucleotides to 10 nm Gold Nanoparticles

Oligonucleotide A and B were conjugated to 10 nm gold particles using a similar protocol to that first described by Mirkin and Letsinger [1]. In detail, 21 μL of oligonucleotide solution (0.156 mM) and 1 mL of 10 nm nanoparticle solution (9.05 nM) we’re mixed and left shaking gently for 16 h at room temperature. Then 127 μL of 1 M NaCl (0.125 vol.) and 127 μL 0.1 M (0.125 vol.) sodium phosphate were added to bring the solution to 0.1 M NaCl and 10 mM phosphate buffer. The solution was then shaken gently for a further 24 h at room temperature. The solution was then centrifuged for 20 min at 10,000 rpm forming a red oil of nanoparticles beneath the clear solution of excess oligonucleotide. The supernatant was carefully removed and the oil resuspended in 1 mL of 0.1 M NaCl/10 mM sodium phosphate buffer. The centrifugation, removal of supernatant and resuspension was repeated two more times. The nanoparticles were finally dissolved in 0.3 M NaCl, 0.01% sodium azide, 10 mM sodium phosphate buffer.

### 3.4. Immobilisation of Hairpin Oligonucleotide A to a Template Stripped Gold Substrate

Gold substrates are cleaved from support immediately before use. A 3 μM solution of the dithiol oligonucleotide in 1.0 M KH_2_PO_4_ is prepared and a 10 μL drop is cast onto the gold substrate. The gold substrates are incubated in a humid environment for 10 h. After which they are washed with UHP H_2_O to remove any non-covalently bound oligonucleotides. A 15 μL drop of 1 mM 6-mercaptohexanol is then cast onto the gold surface. After 1 hour the solution is washed with UHP H_2_O.

### 3.5. Photolysis with Mercury Lamp and Development of Photocleaved Areas with Complementary Oligonucleotide Labelled with Gold Nanoparticles

Gold substrates containing the thiol bound photolabile oligonucleotide were photolysed for 30 min using a mercury lamp (Black Eye bulb, 340 nm). Substrates were placed approximately 15 cm from the bulb of the lamp. Photolysed samples were then washed with water at 90 ºC to remove the non-covalently bound strand of the photocleaved duplex. Photolysed substrates and non photolysed substrates were exposed gold nanoparticles conjugated with complementary oligonucleotides for 3 h at room temperature.

### 3.6. Atomic Force Microscopy (AFM)

To characterize gold substrates with hairpin A immobilised and with gold nanoparticles assembled AFM was utilized. AFM topography analysis was carried out using a. multimode Nanoscope IIIA (Digital Instruments, Santa Barbara, CA) in tapping mode (alternating current) in air using Olympus OMCL-AC240TS. Minimum image modification was carried out, 1^st^ order flattening and plane fitting.

### 3.7. Melting Curves of Hairpin Gold Nanoparticle Conjugates

To monitor nanoparticles conjugated with hairpin A in solution UV spectroscopy was utilised to derive thermal dissociation curves. Melting curves were collected on a JASCO V-650 double beam spectrophotometer fitted with a JASCO ETC-717 peltier system. Samples were placed in a 1 cm path length cuvette and UV and optical absorption spectra were recorded between 20 °C–90 °C with a temperature gradient of 1 °C/min. The absorbance of the buffer was recorded in the reference slot and automatically subtracted from the recorded absorption data. At least two melting experiments were carried out for each complex.

## 4. Conclusions

We have shown for the first time that it is possible to synthesize a photolabile hairpin terminated with a thiol functionality that can be used to conjugate the hairpin to both gold surfaces and nanoparticles. The photocleavage of such a hairpin on gold, yields a surface activated with a single stranded oligonucleotide which can be utilized to direct the assembly of nanoparticles conjugated with a complementary strand. Analysis of photocleaved samples gives nanoparticle coverage of 17.7%; however the nonphotocleaved sample gives coverage of 1.3%, one order of magnitude less. This illustrates the ability of photocleavable hairpins to direct the assembly of nanomaterials on conducting materials. Such nanoengineering of conductive surfaces is of great interest in the areas of biosensors and bionanoelectronics. Also demonstrated for the first time, is that by conjugating a photocleavable hairpin to a gold nanoparticle it is possible to observe a minor DNA interstrand interaction, otherwise hidden, by comparing the thermal dissociations of a hairpin-nanoparticle conjugates at 260 nm and 520 nm. We have also shown that it is possible to permanently alter the physiochemical properties of DNA-nanoparticles by the introduction of a photocleavable group. Indeed for the first time it has been shown that by exposure to UV light, the disassembly of nanoparticle aggregates can be induced. As an interest grows in the controlled assembly of nanoparticles by crosslinking with DNA such results may help in the development of new and interesting materials with a variety of applications. These results may also be of interest in the study of subtle interactions of secondary DNA structures and the detection of important secondary structures of biological relevance.

## Supplementary Material
