# Supplementary material for: Use of Oligonucleotides Carrying Photolabile Groups for the Control of the Deposition of Nanoparticles in Surfaces and Nanoparticle Association
Source: Int J Mol Sci. 2011 Oct 24;12(10):7238–49. doi: 10.3390/ijms12107238 (PMC3211035; doi:10.3390/ijms12107238)
Supplement: Supplementary file 1 [file ijms-12-07238-s001.pdf]

Supplementary Information

Brendan Manning [1,2] and Ramon Eritja [1,2,*]

[1] Institute for Research in Biomedicine, Baldiri Reixac 10, Barcelona E-08028, Spain;
E-Mail: Brendan.manning@gmail.com

[2] Institute for Advanced Chemistry of Catalonia (IQAC), CSIC, CIBER-BBN Networking Centre on Bioengineering, Biomaterials and Nanomedicine, Jordi Girona 18-26, Barcelona E-08034, Spain

* Author to whom correspondence should be addressed; E-Mail: recgma@cid.csic.es;
Tel.: +34-93-403-9942; Fax: +34-93-204-5904.

Received: 10 October 2011 / Accepted: 18 October 2011 / Published: 24 October 2011

Abstract: An oligodeoxynucleotide hairpin containing a photolabile 2-nitrobenzyl group in the loop and terminated with a thiol function was prepared. The photocleavage of such a hairpin on gold yields a surface activated with a single stranded oligonucleotide which can be utilised to direct the assembly of nanoparticles conjugated with a complementary strand. Analysis of photocleaved surfaces gives nanoparticle coverage one order of magnitude higher than nonphotocleaved surfaces. This illustrates the ability of photocleavable hairpins to direct the assembly of nanomaterials on conducting materials. The conjugation of the photocleavable hairpin to a gold nanoparticle allows the observation of intermolecular interactions between hairpins linked in different nanoparticles, by comparing the thermal dissociations of a hairpin-nanoparticle conjugates at 260 nm and 520 nm. We have also shown that it is possible to permanently alter the physiochemical properties of DNA-nanoparticles by the introduction of a photocleavable group. Indeed for the first time it has been shown that by exposure to UV light the disassembly of nanoparticle aggregates can be induced.

Keywords: DNA; gold nanoparticles; photolysis

Figure S1. Tapping mode AFM image of a freshly cleaved template stripped gold surface.

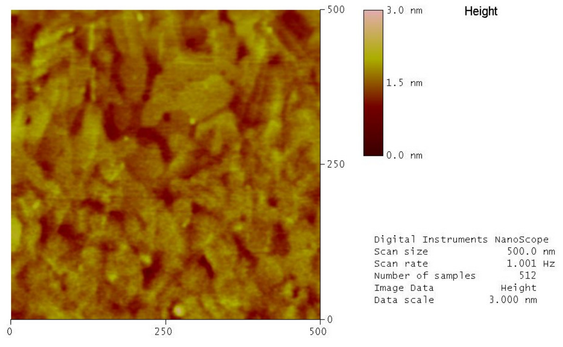

Figure S2. Tapping mode AFM image of hairpin oligonucleotide on a template stripped gold surface.

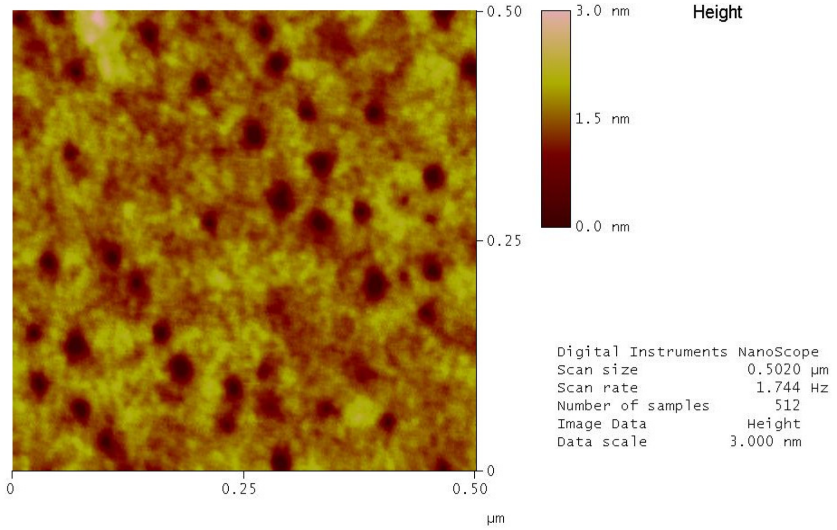

Figure S3. (**a**) Tapping mode AFM images of nanoparticles deposited on a photocleaved hairpin sample and (**b**) nanoparticles deposited on a non photocleaved sample. Below, grayscale images with adjusted threshold of (a) and (b).

(a) (b)

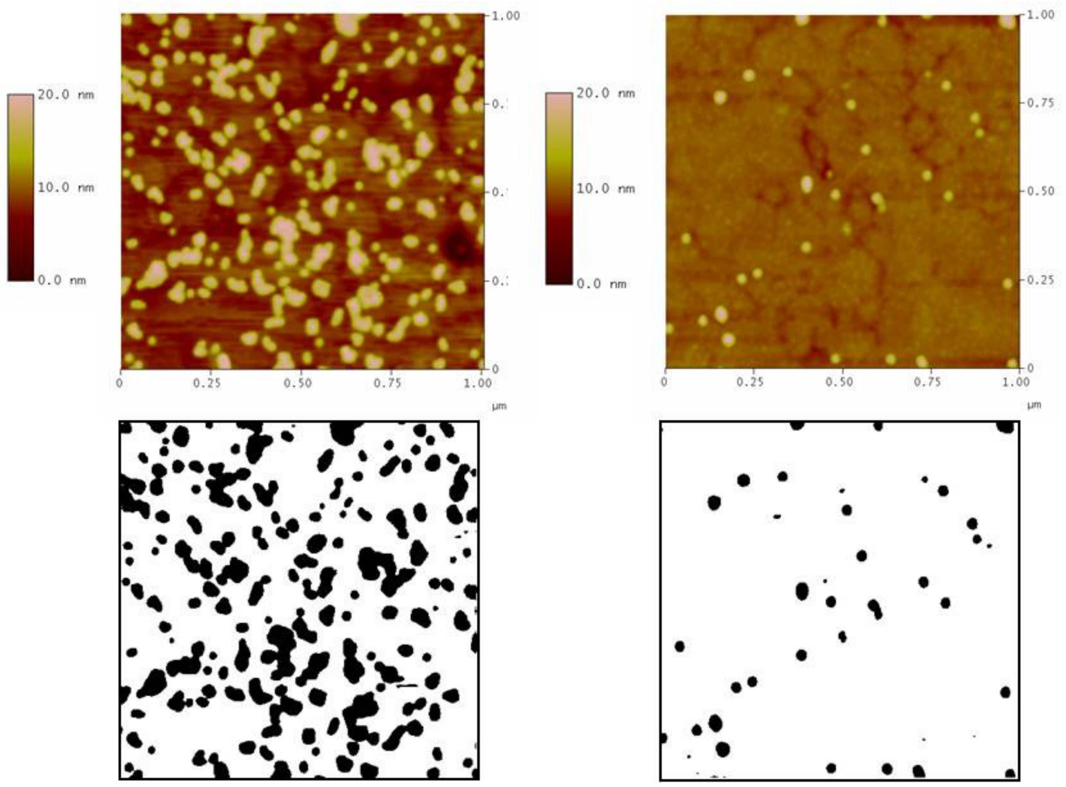

© 2011 by the authors; licensee MDPI, Basel, Switzerland. This article is an open access article distributed under the terms and conditions of the Creative Commons Attribution license (http://creativecommons.org/licenses/by/3.0/).
